# MuscleExpert: a semi-automated tool delivering ImageJ-comparable measurements of muscle thickness, area, and quality

**DOI:** 10.7717/peerj.21757

**Published:** 2026-09-23

**Authors:** Zongpan Li, Li-Qun Zhang, Marcel Bahia Lanza

**Affiliations:** Physical Therapy and Rehabilitation Sciences, University of Maryland Baltimore, Baltimore, MD, United States of America

**Keywords:** Muscle size, Muscle quality, Software, Ultrasound imaging, Muscle thickness, Cross-sectional area, MuscleExpert software

## Abstract

**Background:**

Most image-analysis software programs were not designed specifically for ultrasound-based measurements, resulting in irrelevant general-purpose features that force users to navigate through multiple menus and making the analysis time-consuming and increasing the likelihood of error. The aim of this study was to present and validate the semi-automated MuscleExpert software against the widely used ImageJ by comparing muscle thickness (MT), cross-sectional area (CSA), and muscle quality (MQ-measured as echo intensity).

**Methods:**

Ten volunteers (five males; age: 44.9 ± 23.5 years) participated in this cross-sectional study. Ultrasound images of the tibialis anterior and medial gastrocnemius muscles were acquired using 2D B-mode ultrasonography and analyzed in both software programs. Equivalence of means was evaluated *via* the two-one-sided tests (TOST) procedure; the reliability between means was quantified by an intraclass correlation coefficient (ICC); the precision was expressed as the coefficient of variation (CV) and standard error of measurement (SEM); and agreement was assessed using Bland–Altman plots.

**Results:**

The TOST for MT, CSA, and MQ were within their respective equivalence margins. The ICC was excellent for all measures (MT: ICC = 0.999; CSA: ICC = 0.999; MQ: ICC = 1.000). Precision was similarly high, with CVs of 0.49% (MT), 0.54% (CSA), and 0.33% (MQ), demonstrating minimal variability between the two methods. SEM values were low across all outcomes, corresponding to 0.49% (MT), 0.53% (CSA), and 0.23% (MQ). Finally, the Bland–Altman analysis demonstrated minimal systematic differences and no proportional bias between ImageJ and MuscleExpert for all metrics. The present findings indicate that MuscleExpert and ImageJ produce nearly identical results for MT, CSA, and MQ. These findings support the integration of MuscleExpert into clinical and research workflows, offering a more efficient solution for muscle assessment without compromising measurement integrity.

## Introduction

Two-dimensional (2D) ultrasound (US) imaging is widely used for clinical and research purposes across the health sciences field (Whittaker & Stokes, 2011). Clinicians often record images of organs to assess structural size and tissue quality (Fang & Sidhu, 2020; Imasawa et al., 2012), while researchers and clinicians use B-mode ultrasound to capture images of specific muscles and quantify both muscle size (Lanza et al., 2025) and muscle quality (*e.g.*, echo intensity) (Stock & Thompson, 2021). The accuracy and consistency of ultrasound-derived measurements can vary depending on the analysis software and user workflow (Cain & Haque, 2008). To ensure reliable and reproducible results, as well as decreased time for data analysis, there is a clear need for software specifically designed for ultrasound image analysis.

Most image-analysis software, such as Horos, OsiriX, and ImageJ, were not specifically designed for ultrasound-based muscle morphology measurements. Consequently, they contain numerous general-purpose features that are irrelevant to this application and force users to navigate through multiple menus and dialogs. In many cases, the overwhelming amount of tools can prolong data analysis and may lead users to select the wrong functions, resulting in measurement errors (Lateef et al., 2024). Thus, this may be the reason for researchers to develop their own custom image-analysis software and/or scripts (Mul et al., 2018; Stein et al., 2023). However, these tools are rarely shared with the wider community, and there is no assurance that the measurements they produce will agree with those generated by established, widely used programs. Consequently, it is possible that measurements derived from such tools differ meaningfully from those obtained using standard, well-documented platforms—introducing potential bias or error into published findings.

Muscle size and quality are types of measurements that are constantly performed by different research groups using ultrasound (Lanza et al., 2025; Franchi et al., 2018; Abe et al., 2000; Chang et al., 2019; Harris-Love et al., 2018; Rock et al., 2021). The image analysis to extract the values from these measurements can be time-consuming. For reference, a typical analysis on ImageJ software to extract both measurements (muscle size and quality) proceeds through the following steps: (1) loading the raw ultrasound image; (2) opening the calibration tool; (3) drawing a reference line over a region of known length (*i.e.,* the probe’s field of view); (4) entering the corresponding distance to set the image scale; (5) opening brightness/contrast settings (not always required); (6) adjusting brightness and/or contrast (not always required); (7) applying image filters to enhance visualization of superficial and deep aponeuroses (not always required); (8) selecting the muscle-thickness measurement tool; (9) tracing a line between the superficial and deep aponeuroses of the muscle; (10) recording the value; (11) selecting the polygon tool to draw the region of interest (ROI) for echo-intensity analysis; (12) tracing the ROI; (13) extracting the mean echo intensity value; and (14) saving or exporting the measurement data. Switching between these tools for each task not only prolongs the process but also increases the risk of inconsistency and user error. Furthermore, such multi-step workflows demand considerable training, and the reliance on user judgment at multiple decision points has been associated with modest inter-rater reliability when different researchers analyze the same images (Carr et al., 2021).

Recently, the MuscleExpert software was released freely on Zenodo (Bahia Lanza, 2025). This semi-automatic Python-based program was designed to reduce analysis time by minimizing manual steps and automatically selecting the appropriate tools based on established muscle-morphology protocols across different research groups (Franchi et al., 2018; Lanza et al., 2022; Larsen et al., 2025; Saito et al., 2024; Nishihara et al., 2024). Unlike ImageJ, MuscleExpert streamlines analysis into three steps: (1) loading the raw US image; (2) cropping the image of the muscle; (3) drawing the ROI. By collapsing more than a dozen manual operations into just these three actions, MuscleExpert might reduce analysis time by approximately 80% (see File S1) and may substantially reduce the risk of tool-selection errors, ensuring faster, and potentially more consistent measurements across users. However, the reproducibility and reliability of MuscleExpert have not been demonstrated, nor has its agreement with other well-known software.

Therefore, the aim of this study is to assess the agreement and reliability of the semi-automated MuscleExpert software with ImageJ, a widely used, freely available image-analysis platform extensively applied in ultrasound-based muscle research, by comparing muscle thickness (MT), cross-sectional area (CSA), and muscle quality (MQ-measured as echo intensity) metrics to assess agreement and reliability between the two approaches. It was hypothesized that the MuscleExpert software would demonstrate excellent agreement and reliability with ImageJ, thereby supporting its adoption as a tool for ultrasound image analysis.

## Materials and Methods

### Subjects

This cross-sectional study was approved by the University of Maryland Institutional Review Board (HP-00109908). A convenience sample of 10 participants (five males, five females; 18 to 85 years old; age: 44.9 ± 23.5 years; height: 1.65 ± 0.8 m; body mass: 67.3 ± 16.4 kg) provided written informed consent before their participation. All participants, who had no history of neurological or muscular disorders, nor serious injuries or surgery of the lower extremity, volunteered to participate in the study. Portions of this text were previously published as part of a preprint (Lanza, 2025).

### Measurements and procedures

**Figure 1:**
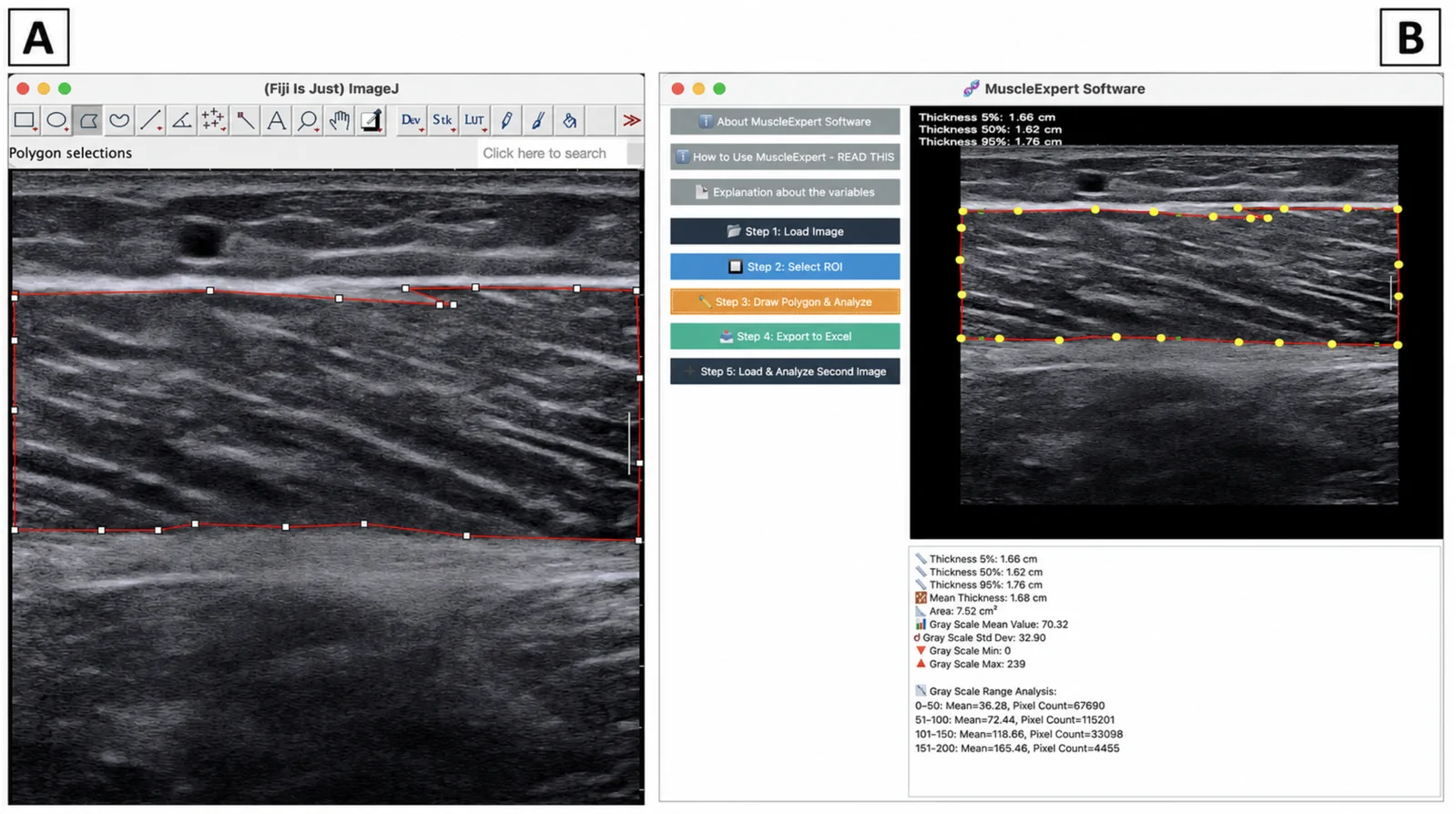
Example of a cross-sectional area measurement from the medial gastrocnemius from one participant on (A) ImageJ software and (B) MuscleExpert software.

Participants remained seated (knees at 90° of flexion) and rested for 10 min before ultrasound measurements. Ultrasound recordings and analysis were performed for tibialis anterior (TA) and medial gastrocnemius (MG) muscles. The anterior and posterior regions of the dominant leg were marked to identify the reference points for the ultrasound image acquisition. The dominant limb was defined by the question: which foot would you use to kick a ball (Lanza et al., 2022). Skin marks were made at 1/3 on the line between the tip of the fibula and the tip of the medial malleolus for the TA muscle, while placed on the most prominent bulge of the muscle for MG. Two ultrasound images per site were obtained using B-mode ultrasonography (Aixplorer, Supersonic Imagine, Aix-en-Provence, France) with a SuperLinear 5–18 MHz frequency probe, gain of 62 dB, a depth of 6.0 cm. An example of US images can be seen in Fig. 1. All images were acquired by the same evaluator (Z.L.), with 10 years of experience in musculoskeletal ultrasound imaging. Probe pressure was kept minimal throughout image acquisition and was continuously monitored by verifying the absence of visible skin or tissue compression within the image.

### Data analysis

For each muscle, twenty images were generated, yielding a total of forty images for analysis. Image analysis was performed using two software programs: ImageJ software, version 1.53a (National Institutes of Health, Bethesda, MD, USA) and MuscleExpert software, version 1.0. The image analysis comprised two stages. In the first stage, each image was analyzed twice in each program (ImageJ and MuscleExpert), and the mean of those two readings was calculated for each program. In the second stage of analysis, the same procedure was repeated. The grand mean, computed as the average of the two stages, served as the final measurement for all subsequent analyses. Repeating measurements within and between sessions and then averaging them has been shown to reduce random error and to allow formal assessment of intra-rater consistency using intraclass correlation coefficients (Weir, 2005; Atkinson & Nevill, 1998). Moreover, this design enables the detection of any systematic bias (*e.g.*, learning or fatigue effects) between stages, yielding more precise and trustworthy estimates.

Ultrasound images were exported from the imaging device in JPEG format and imported into both software programs. In ImageJ, MT was measured as the straight-line distance between the superficial and deep aponeuroses on each image, while muscle quality (mean echo intensity value) was determined by outlining the ROI in the TA and MG muscles with the polygon tool (Lateef et al., 2024) and recording the average echo intensity value within that area. In MuscleExpert, the polygon tool was used to delineate muscle ROI, but both MT and mean echo intensity were calculated automatically from the drawn region. In both software programs, MT was measured at 50% of the image size as previously (Lanza et al., 2025). In MuscleExpert, the image processing steps followed the three-step sequence described in the Introduction: loading the image, cropping the muscle region, and drawing the ROI, from which all variables were extracted automatically. No additional grayscale normalization was applied, nor was it required: both software programs analyzed the exact same image files, meaning the underlying pixel intensity values were identical across programs. Grayscale normalization is only necessary when comparing measurements derived from images acquired under different conditions; since both programs processed the same image, pixel values were inherently equivalent.

### Statistical analysis

Statistical analyses were conducted in Python using the Visual Studio Code IDE (Microsoft Corporation, version 1.90.2), and an alpha level of 0.05 was applied to determine statistical significance. Data from the two muscles were pooled because our goal was to compare software performance rather than muscle-specific differences. All variables were assessed for normality using the Shapiro–Wilk test. Equivalence of means was then evaluated *via* the two-one-sided tests (TOST) procedure (Lakens, 2017) with a priori equivalence margins (Δ) of ±0.25 cm for thickness, ±0.65 cm^2^ for area, and ±9 arbitrary units (au.). Two one-sided paired *t*-tests were performed and equivalence concluded if both *p*-values were <0.05 and the 90% confidence interval for the mean difference lay entirely within [−Δ, +Δ] (Lakens, 2017). Margins for MT (Lanza et al., 2025; McCreesh & Egan, 2011; Yuguchi et al., 2020; Wang, Hu & Tian, 2018; Arts et al., 2010) and MQ (as echo intensity) (Yuguchi et al., 2020; Arts et al., 2010; Yao et al., 2023; Caresio et al., 2015) in both muscles were calculated from prior studies. For each variable, the range of typical between-measurement differences reported across these studies was identified for each muscle, and the most conservative threshold, that is, the smallest margin, not exceeding 8% of the mean, was selected to ensure the most rigorous criterion for equivalence. Because measuring CSA on longitudinal US images is not standard practice, a conservative 8% margin was applied based on our own ImageJ reference data, consistent with the approach used for the other variables. Absolute agreement was quantified by an intraclass correlation coefficient (ICC_(3,1)_), interpreted as weak (<0.4), moderate (0.4–0.59), good (0.6–0.74), and/or excellent (0.75–1.0) (Cicchetti, 1994). Precision was expressed as the coefficient of variation (CV = [within-subject SD/overall mean] ×100%). The standard error of measurement (SEM) was computed using the classical test theory formula: SEM = SD ×√(1–ICC), where SD represents the pooled standard deviation of the two measurement methods and ICC refers to the absolute agreement intraclass correlation coefficient (ICC_(3,1)_). SEM was further expressed as a percentage of the overall mean to provide a scale-free index of absolute error. Agreement across the measurement range was further explored using Bland–Altman plots, reporting mean bias and 95% limits of agreement (mean difference ±1.96 × SD).

**Figure 2:**
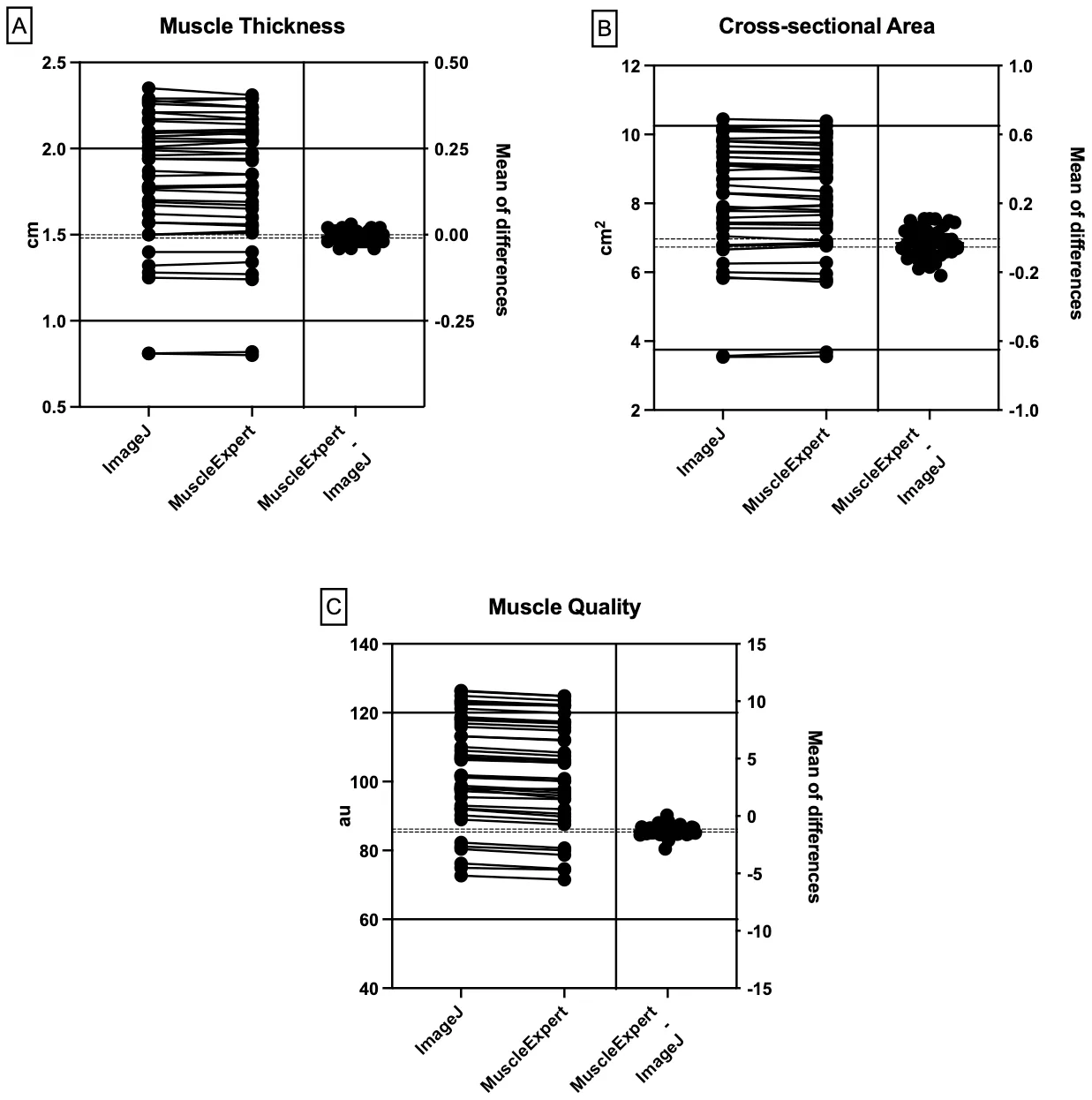
Mean values of each individual measure on ImageJ and MuscleExpert software programs and the mean-difference results for (A) muscle thickness, (B) cross-sectional area, and (C) muscle quality. Solid black lines denote the pre-specified lower (–Δ ) and upper (+Δ ) equivalence margins used in the two-one-sided tests (TOST). Dashed lines indicate the lower and upper bounds of the 90% confidence interval around the observed mean difference. Equivalence is achieved when the entire dashed interval lies within the solid black margins.

## Results

**Figure 3:**
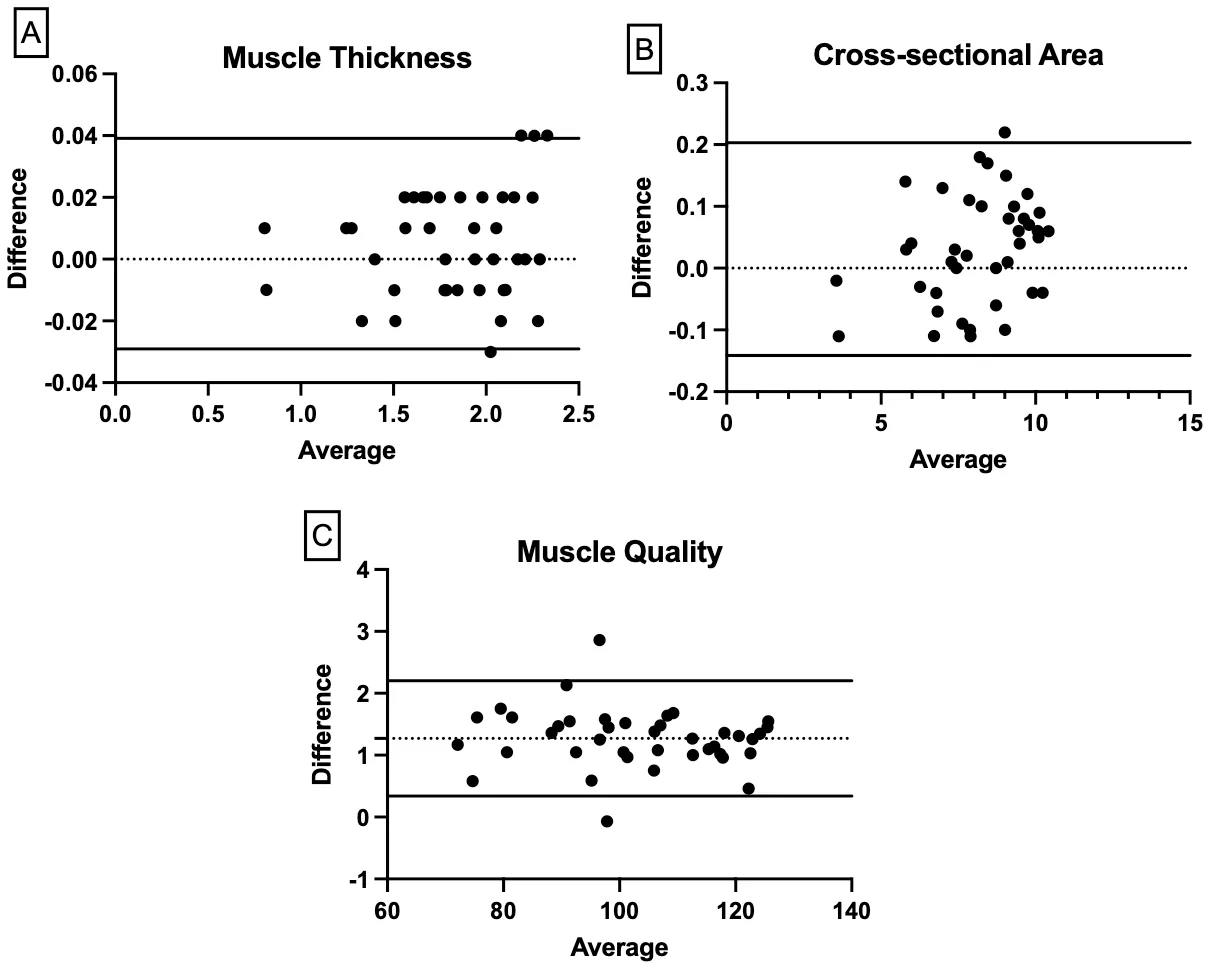
Bland-Altman plots for the agreement between MuscleExpert and ImageJ for muscle thickness (A), cross-sectional area (B), and muscle quality (C), Solid lines represent the upper and lower limits of agreement (±1.96 SD). The dotted line represents the mean bias.

The TOST for MT yielded a mean difference of 0.005 cm (90% CI [0.0004–0.010] cm; p_lower_ <  0.0001, p_upper_ < 0.0001), which lies entirely within the ±0.25 cm margin, Fig. 2A. CSA differences averaged 0.031 cm^2^ (90% CI [0.007–0.054] cm^2^; p_lower_ < 0.0001, p_upper_ < 0.0001; Fig. 2B) and MQ differences averaged 1.27 au. (90% CI [1.143–1.397] au.; p_lower_ < 0.0001, p_upper_ <  0.0001; Fig. 2C), both comfortably within their respective equivalence margins. Absolute agreement was excellent for all measures (MT: ICC = 0.999; CSA: ICC = 0.999; MQ: ICC = 1.000), Table 1. Precision was similarly high, with CVs of 0.49% for MT, 0.54% for CSA, and 0.33% for MQ, demonstrating minimal variability between the two methods. SEM values were low across all outcomes, corresponding to 0.008 cm (0.49 %) for MT, 0.04 cm^2^ (0.53 %) for CSA, and 0.36 au. (0.23 %) for MQ, Table 1. Bland–Altman analysis demonstrated minimal systematic differences and no proportional bias between ImageJ and MuscleExpert for all three metrics. For MT bias was 0.0052 cm and 95% limits of −0.0296 to 0.0401 cm (slope = 0.0084, *p* = 0.2673), Fig. 3A. For CSA the bias was 0.0309 cm^2^ (95% limits −0.1396 to 0.2014 cm^2^) and a non-significant slope of 0.0154 (*p* = 0.0610), Fig. 3B. Lastly, MQ bias was 1.2701 au., with limits of 0.3379 to 2.2023 au. and slope −0.0037 (*p* = 0.4654), Fig. 3C.

**Table 1:** Intraclass correlation coefficients (ICC), coefficients of variation (CV), and standard error of measurement (SEM) for MuscleExpert versus ImageJ software.

**Variable**	**ICC (95% CI)**	**CV (%)**	**SEM (95% CI)**	**SEM (%)**
Muscle thickness	0.999 (0.998–1.000)	0.49	0.008 (0.0072–0.0103) cm	0.49%
Cross-sectional area	0.999 (0.999–1.000)	0.54	0.04 (0.03–0.05) cm^2^	0.53%
Muscle quality	1.000 (0.999–1.000)	0.33	0.23 (0.20–0.26) au.	0.23%

## Discussion

Since custom image-analysis pipelines are often neither published nor benchmarked against established standards, there is no guarantee their results align with those produced by validated, widely adopted software. Thus, the first aim of this study was to assess the agreement and reliability of the semi-automated MuscleExpert software against ImageJ by comparing MT, CSA, and MQ measurements for agreement and reliability. The present results support our hypothesis that the MuscleExpert software demonstrates excellent agreement and reliability with ImageJ. It is important to note that this study establishes software-to-software agreement rather than measurement validity; validity would require comparison against a criterion standard independent of both programs, such as direct anatomical measurement. In summary, the MuscleExpert software offers a reliable, efficient alternative to ImageJ for quantitative ultrasound assessment of muscle morphology.

The test of equivalence confirmed that the differences in MT, CSA, and MQ between the two methods fell entirely within the predefined equivalence margins, supporting the practical equivalence of the two methods. Importantly, highly conservative margins were adopted for all variables. This suggests that MuscleExpert can be considered a comparable alternative to ImageJ for these measurements. In addition to that, the exceptionally high intraclass correlation coefficients (ICCs ≥ 0.999) and low coefficients of variation (CVs < 1%) further support the precision and consistency of MuscleExpert, indicating minimal variability and excellent reproducibility across all three muscle morphology measurements. SEMs were also consistently low across all outcomes, with values below 0.05% of the pooled mean, further demonstrating the high accuracy of MuscleExpert compared to ImageJ. These minimal SEM values reinforce the notion that the software introduces minimal measurement error. Bland–Altman analyses reinforced these findings by showing minimal systematic bias and no evidence of proportional bias between the two methods. The narrow limits of agreement and non-significant regression slopes across all metrics suggest that MuscleExpert performs comparably to ImageJ across the full range of values. Notably, the MQ metric, often more susceptible to variability due to echo intensity interpretation (Stock & Thompson, 2021), showed the highest precision (CV = 0.33%) and near-perfect agreement (ICC = 1.000), noting that an ICC of 1.000 is uncommon in biological measurements. This likely reflects both the limited between-subject variability in this convenience sample and the within-method variance reduction introduced by the repeated-measures averaging design. Thus, these results should be interpreted with appropriate caution, highlighting the robustness of MuscleExpert’s echo intensity analysis. These findings collectively support the use of MuscleExpert software as a reliable and efficient tool for muscle assessment, offering potential advantages in clinical and research settings where automation and scalability are increasingly important.

Each program presents both advantages and limitations. MuscleExpert is specifically engineered for ultrasound imaging in health-science disciplines (*e.g.*, exercise physiology, physical therapy), offering a minimalist interface designed to minimize tool-selection errors and reduce configuration time. In contrast, ImageJ is an open-source, extensible environment with comprehensive filters, plugins, scripting support (Java, Python), batch-processing pipelines, and detailed metadata extraction, though its extensive feature set may require a longer learning period. Consequently, MuscleExpert may be better suited for rapid, standardized analyses, whereas ImageJ may better serve users requiring deep customization and automation. From a practical standpoint, the present findings suggest that, under conditions similar to those studied, MuscleExpert may produce measurements comparable to those obtained with ImageJ, potentially allowing data from both platforms to be used interchangeably. For researchers currently using ImageJ, no immediate change in practice may be warranted; however, for groups initiating new projects or seeking to reduce user-dependent variability, MuscleExpert may offer a more accessible and time-efficient alternative. Its streamlined workflow may also support greater standardization across research groups, though broader validation across diverse samples, operators, and imaging conditions is needed before definitive recommendations can be made.

Limitations and strengths of this study should be acknowledged. First, the sample size was small (*n* = 10) and comprised a convenience sample with a wide age range (18–85 years), which limits the statistical power of the findings and may reduce their generalizability to more homogeneous clinical or research populations. Second, all image analyses were performed by a single operator; therefore, inter-rater reliability was not assessed, and the extent to which these findings generalize to different analysts remains unknown. A notable strength of this study is the inclusion of two anatomically and morphologically distinct muscles, the tibialis anterior and the medial gastrocnemius, analyzed as a pooled dataset. This design reflects an important conceptual consideration: the present study aimed to evaluate whether the same image analysis method, applied across two different software platforms, produces equivalent results, rather than to validate either software for a specific muscle group. The analytical steps used to extract MT, CSA, and echo intensity from an ultrasound image are methodologically consistent regardless of the anatomical structure being examined. Combining measurements from two distinct muscles therefore offers a more comprehensive assessment of equivalence at the methodological level. The consistent agreement observed across both muscles supports the conclusion that the equivalence between MuscleExpert and ImageJ is due to the analytical method, independent of the specific muscle analyzed. In addition, in a partial analysis of image analysis time, MuscleExpert appeared to require considerably less time per image compared to ImageJ, suggesting a potential time advantage that, if confirmed in larger samples, could translate to meaningful time savings in research and clinical workflows (see  File S1). Finally, the study was conducted under standardized laboratory conditions using a single imaging protocol and ultrasound device; performance of MuscleExpert under different probe frequencies, image depths, or acquisition settings has not been evaluated and warrants future investigation.

In conclusion, the findings indicate that MuscleExpert and ImageJ produce nearly identical results for MT, CSA, and MQ. The equivalence testing, high ICC, low CVs and SEM values, and Bland–Altman analyses collectively demonstrate that MuscleExpert provides measurements that are statistically equivalent and practically comparable to those obtained with ImageJ. These findings support the integration of MuscleExpert into clinical and research workflows, offering a more efficient solution for muscle assessment without compromising measurement integrity.

## Supplemental Information

10.7717/peerj.21757/supp-1 Supplemental Information 1 Methods and image-level data Compares the time required to measure muscle thickness, cross-sectional area, and echo intensity in 20 ultrasound images using MuscleExpert and ImageJ, including statistical results and estimated time savings for the full study dataset

10.7717/peerj.21757/supp-2 Supplemental Information 2 Data

10.7717/peerj.21757/supp-3 Supplemental Information 3 STROBE checklist

## Additional Information and Declarations

### Data Availability

The following information was supplied regarding data availability: The data is available in the Supplemental Files.
