# Supplementary material for: MuscleExpert: a semi-automated tool delivering ImageJ-comparable measurements of muscle thickness, area, and quality
Source: PeerJ. 2026 Sep 23;14:e21757. doi: 10.7717/peerj.21757 (PMC13615733; doi:10.7717/peerj.21757)
Supplement: Supplementary file 2 [file peerj-14-21757-s001.docx]

**Supplemental File**

**Methods**

A trained evaluator recorded the time required to fully analyze each of 20 ultrasound images using MuscleExpert (version 1.0) and ImageJ (version 1.53a; National Institutes of Health, USA). Timing was initiated with a stopwatch at the moment the evaluator began the image analysis in each software program and was stopped upon completion of all measurements for that image. The same 20 ultrasound images were analyzed in both software programs by the same evaluator. For MuscleExpert, the timed analysis covered the extraction of all variables automatically provided by the software: muscle thickness at three anatomical locations (proximal, middle, and distal), cross-sectional area, and echo intensity. For ImageJ, the identical set of variables was extracted manually to ensure a fair and equivalent comparison, specifically, muscle thickness at the same three locations, cross-sectional area, and echo intensity. A paired-samples t-test was used to compare the mean analysis time per image between the two software programs. Descriptive statistics including mean, standard deviation (SD), and coefficient of variation (CV) were calculated for each program. Effect size was estimated using Cohen’s d for paired data.

**Data**

Table S1. Time (in seconds) required to analyze each ultrasound image using MuscleExpert and ImageJ. CV = coefficient of variation.

| **Image #** | **MuscleExpert (s)** | **ImageJ (s)** |
| --- | --- | --- |
| 1 | 126 | 650 |
| 2 | 118 | 640 |
| 3 | 150 | 540 |
| 4 | 118 | 701 |
| 5 | 180 | 745 |
| 6 | 145 | 667 |
| 7 | 165 | 687 |
| 8 | 179 | 550 |
| 9 | 166 | 690 |
| 10 | 123 | 520 |
| 11 | 134 | 730 |
| 12 | 143 | 834 |
| 13 | 146 | 559 |
| 14 | 133 | 712 |
| 15 | 171 | 674 |
| 16 | 154 | 556 |
| 17 | 117 | 678 |
| 18 | 128 | 781 |
| 19 | 141 | 578 |
| 20 | 128 | 612 |
| **Mean ± SD** | **143.3 ± 20.4** | **655.2 ± 85.8** |
| **CV (%)** | **14.2** | **13.1** |

**Results**

MuscleExpert required significantly less time per image (143.3 ± 20.4 s) compared to ImageJ (655.2 ± 85.8 s), representing a 78.1% reduction in analysis time per image (paired t-test: t(19) = −25.71, p < 0.001; Cohen’s d = 5.75). Coefficients of variation were similar between programs (MuscleExpert: 14.2%; ImageJ: 13.1%), indicating comparable intra-program consistency in analysis duration. The effect size (d = 5.75) reflects an exceptionally large and consistent difference in analysis speed between the two programs.

**Estimated Time Savings for the Full Study**

The main study analyzed a total of 40 ultrasound images (20 per muscle: tibialis anterior and medial gastrocnemius). Each image was analyzed four times per software program, twice within each of two separate sessions, yielding 160 individual image analyses per program. Extrapolating from the per-image timing data collected in this supplemental analysis, the estimated total analysis time for the full dataset was approximately 382 minutes (6.4 hours) using MuscleExpert, compared to approximately 1,747 minutes (29.1 hours) using ImageJ. This represents an estimated saving of approximately 1,365 minutes (~22.8 hours) across the full study by using MuscleExpert instead of ImageJ.
